# Application of Dynamic ^18^F-FDG PET/CT for Distinguishing Intrapulmonary Metastases from Synchronous Multiple Primary Lung Cancer

**DOI:** 10.1155/2022/8081299

**Published:** 2022-06-30

**Authors:** Weize Lv, Min Yang, Hongcheng Zhong, Xiaojin Wang, Shuai Yang, Lei Bi, Jianzhong Xian, Xiaofeng Pei, Xinghua He, Ying Wang, Zhong Lin, Qingdong Cao, Hongjun Jin, Hong Shan

**Affiliations:** ^1^Guangdong Provincial Key Laboratory of Biomedical Imaging, The Fifth Affiliated Hospital, Sun Yat-sen University, Zhuhai, Guangdong Province 519000, China; ^2^Department of Medical Oncology, The Fifth Affiliated Hospital, Sun Yat-sen University, Zhuhai, Guangdong Province 519000, China; ^3^Department of Cardiothoracic Surgery, The Fifth Affiliated Hospital, Sun Yat-sen University, Zhuhai, Guangdong Province 519000, China; ^4^Department of Ultrasound, The Fifth Affiliated Hospital, Sun Yat-sen University, Zhuhai, Guangdong Province 519000, China; ^5^Nuclear Medicine Department, The Fifth Affiliated Hospital, Sun Yat-sen University, Zhuhai, Guangdong Province 519000, China; ^6^Interventional Department, The Fifth Affiliated Hospital, Sun Yat-sen University, Zhuhai, Guangdong Province 519000, China

## Abstract

It has been a big challenge to distinguish synchronous multiple primary lung cancer (sMPLC) from primary lung cancer with intrapulmonary metastases (IPM). We aimed to assess the clinical application of dynamic ^18^F-FDG PET/CT in patients with multiple lung cancer nodules. We enrolled patients with multiple pulmonary nodules who had undergone dynamic ^18^F-FDG PET/CT and divided them into sMPLC and IPM groups based on comprehensive features. The SUV_max_, fitted *K*_*i*_ value based on dynamic scanning, and corresponding maximum diameter (*D*_max_) from the two largest tumors were determined in each patient. We determined the absolute between-tumor difference of SUV_max_/*D*_max_ and *K*_*i*_/*D*_max_ (ΔSUV_max_/*D*_max_; Δ*K*_*i*_/*D*_max_) and assessed the between-group differences. Further, the diagnostic accuracy was evaluated by ROC analysis and the correlation between ΔSUV_max_/*D*_max_ and Δ*K*_*i*_/D_max_ from all groups was determined. There was no significant difference for ΔSUV_max_/*D*_max_ between the IPM and sMPLC groups, while the IPM group had a significantly higher Δ*K*_*i*_/D_max_ than the sMPLC group. The AUC of Δ*K*_*i*_/*D*_max_ for differentiating sMPLC from IPM was 0.80 (cut-off value of *K*_*i*_ = 0.0059, sensitivity 79%, specificity 75%, *p* < 0.001). There was a good correlation (Pearson *r* = 0.91, 95% CI: 0.79-0.96, *p* < 0.0001) between ΔSUV_max_/*D*_max_ and Δ*K*_*i*_/*D*_max_ in the IPM group but not in the sMPLC group (Pearson *r* = 0.45, *p* > 0.05). Dynamic ^18^F-FDG PET/CT could be a useful tool for distinguishing sMPLC from IPM. *K*_*i*_ calculation based on Patlak graphic analysis could be more sensitive than SUV_max_ in discriminating IPM from sMPLC in patients with multiple lung cancer nodules.

## 1. Introduction

Lung cancer is among the most common cancers and the leading cause of cancer-related death worldwide [1]. Given the advanced diagnostic and surveillance methods, as well as the increasing aging population, there has been a recent increase in the incidence of synchronous multiple primary lung cancer (sMPLC). It is crucial to discriminate sMPLC from primary lung cancer with intrapulmonary metastases (IPM) due to their varying clinical staging, treatment strategies, management, and prognosis. Patients with sMPLC are staged separately and generally treated with curative surgical resection because of early stage and favorable prognosis while those with IPM in advanced stages receive chemotherapy or radiotherapy with palliative intent [2].

Differential diagnosis between sMPLC and advanced lung cancer is traditionally based on conventional histopathologic features. These include location, morphology, histologic type, time interval, lymphatic invasion, metastases, and clinical manifestation, as described by the Martini and Melamed criteria published in 1975 [3], which was subsequently modified by the American College of Chest Physicians (ACCP) [2]. However, this traditional diagnostic process often involves an overlap in a significant proportion of cases, especially for tumors with a similar morphology or histotype, which impedes the determination of whether they share the same clone origin. Moreover, some patients cannot undergo preoperative histological examination or relative surgery. Further, some tumors cannot be sampled and tested pathologically if the patient has a poor physical condition, including limited cardiopulmonary reserves.

Novel molecular and genomic analyses, including biomarker assessment (driver gene mutations) [4], array comparative genomic hybridization [5], shallow whole-genome sequencing [6], and TP53 mutation analysis [7], can define the between-lesion correlation. These molecular biological techniques allow the detection and analysis of specific molecular markers or mutation sites to determine the heterogenicity of two cancer foci. However, given the substantial misclassification rate, which results from limited sensitivity, stabilization, repeatability, and economic benefits, techniques that could be able to precisely define sMPLC or IPM have not been widely applied in clinic [8].

Preoperative differentiation of primary tumors from metastases is more clinically pivotal than postoperative differentiation. Moreover, preoperative imaging examination plays a significant role. However, there is limited information regarding the imaging and metabolic characteristics of multiple lung cancer nodules [8]. Pretreatment chest high-resolution computed tomography (HRCT) is a conventional and cost-effective means of preliminary diagnosis. Since distinguishing IPM from MPLC is empirically based on the morphological pulmonary nodule features, it is difficult to obtain a definitive diagnosis of a primary tumor or metastasis solely based on CT characteristics. Moreover, according to the ACCP clinical practice guidelines, ^18^F-fluorodeoxyglucose positron emission tomography/computed tomography (^18^F-FDG PET/CT) plays a significant role in guiding clinical decisions and is a recommended standard workflow for patients with potentially curable lung cancer [2]. ^18^F-FDG PET/CT has been extensively used in patients with lung cancer for diagnosis [9], staging [10], prediction (i.e., monitoring therapy response) [11], and prognosis [12]. These mentioned applications indicate that ^18^F-FDG PET could be used to identify early-stage sMPLC, which involves multiple pulmonary sites. Several heterogeneous methods could be used to determine ^18^F-FDG uptake. Dynamic PET acquisition, immediately starting from radiotracer injection, measures drug activity change over time. Dynamic PET including multiple frames usually requires long image acquisition time (for ^18^F-FDG, generally lasting for 60 minutes) and only allows the assessment of one FOV. Generally speaking, the radioactivity concentration of radiotracer in blood and tissue will be changing most rapidly early after injection; therefore, most dynamic PET acquisition has finer time frames at early time points and wider time frames at later time points [13]. On the other hand, static PET acquisition is a common clinical PET acquisition mode. After radiotracer injection for a period of time, when the physiological metabolism or binding in vivo is stable, the multiple-FOV and single frame static emission scan is acquired. For ^18^F-FDG, static emission scan lasting for 10-15 minutes is usually started at 45-60 minutes postinjection. In routine clinical practice, the standardized uptake value (SUV) based on static PET scanning, which is a simple semiquantitative index that reflects the metabolic activity of tumor lesions, is widely applied in PET imaging evaluation. SUV is associated with several tumor characteristics, including histopathological subtypes [14]; tumor proliferation [14], differentiation [15], and aggressiveness [16]; and tumor stage, recurrence, and survival [17, 18]. However, the SUV outcome is affected by several factors, including the image acquisition time [19], acquisition mode [20], reconstruction mode [21], serum glucose and insulin levels [22, 23], and positive contrast agent [24]. Theoretically, Patlak graphic analysis based on dynamic PET scanning is the most classical fully quantitative measure of glucose metabolism that involves irreversible trapping. It calculates the ^18^F-FDG net influx rate constant (i.e., uptake rate constant, *K*_*i*_) through linear graphical data fitting [25, 26]. Being a linear kinetic model, Patlak analysis lacks noise amplification and is independent of uptake time and changes in plasma FDG clearance [27, 28].

Since sMPLC with a separate clonal origin often indicates early-stage and less than two-year interval tumorigenesis, we hypothesized that there would be the same SUV or *K*_*i*_ for the initial and second primary lung cancers. Since IPM with an identical clonal origin often indicates late-stage tumorigenesis, we assumed that the SUV or *K*_*i*_ of the primary lung cancer was larger than pulmonary metastases. Moreover, it remains to be assessed whether *K*_*i*_ calculation through Patlak graphic analysis has values for differentiating sMPLC from IPM in patients with multiple lung cancer nodules since it provides more robust dynamic information and accurate ^18^F-FDG metabolism quantification.

This study is aimed at assessing the differential diagnostic ability of dynamic ^18^F-FDG PET scan in patients with lung cancer involving multiple pulmonary sites. This could be able to provide a novel method for sMPLC diagnosis in clinic.

## 2. Materials and Methods

### 2.1. Patients

**Figure 1:**
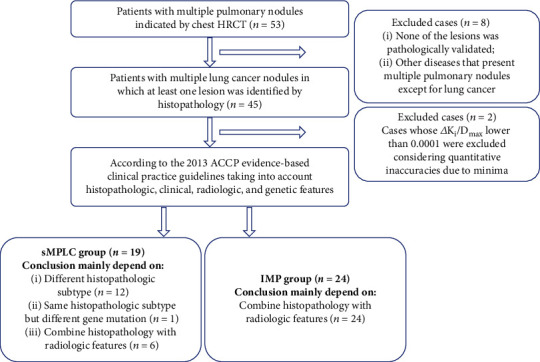
Diagram of case inclusion and exclusion.

In our center, firstly, all first-visit patients with related symptoms underwent preliminary screening using chest HRCT. Then, other examinations mainly including serologic examination, tumor-associated antigen, brain magnetic resonance imaging, and cervical and abdominal CT scan further clarified the general condition and clinical TNM classification. Fifty-three patients with multiple pulmonary nodules regardless of extrapulmonary metastases received dynamic ^18^F-FDG PET scans and had not received any medical treatment before this. After careful preoperative evaluation based on above examination, twenty-seven suitable patients (stage I, stage II, and partial stage IIIA) without medical contraindications received radical surgical resection. Partial inoperable patients (stage IV and partial stage IIIA) were followed by CT-guided percutaneous lung puncture biopsy, bronchoscopic biopsy, or superficial lymph node biopsy mainly based on tumor location. What calls for special attention was that two cases whose △*K*_*i*_/*D*_max_ lower than 0.0001 were excluded considering quantitative inaccuracies due to minima. Finally, this study enrolled forty-three patients with multiple lung cancer nodules (either suspected or proven by biopsy/resection). Based on histopathologic, clinical, radiologic, and genetic features, two experienced oncologists divided those patients into the sMPLC (19 cases) and IPM groups (24 cases) reference to the ACCP evidence-based clinical practice guidelines [2]. That is, the division criteria of sMPLC are (1) same histology, tumor in different lobe as primary, and no N2 and N3 involvement, and no systemic metastases; (2) or different histology, molecular genetic characteristics or arising from a separate focus of carcinoma in situ [2]. The division criteria of IPM are (1) same histology and multiple systemic metastases; (2) or same histology in different lobe and presence of N2 and N3 involvement; (3) or <2-year interval [2]. The inclusion criteria were (1) patients whose preliminary chest HRCT showing multiple pulmonary nodules; (2) patients who performed dynamic ^18^F-FDG PET/CT for pulmonary nodules; (3) at least two lesions were identified as lung cancers based on histopathologic, clinical, radiologic, and genetic features. The exclusion criteria were (1) no pathological validation; (2) having other diseases that present multiple pulmonary nodules; (3) known history of other malignant diseases; (4) diabetes and/or severe cardiovascular disease.  Figure 1 presents the flowchart for case inclusion. Specifically, we only included one lesion pair (two lesions: the largest and second-largest lesion) per patient for simplifying comparisons and calculations, especially for patients with more than two lesions in the lung. Based on the anatomic sites of both lesions, two tumor nodules were subgrouped as follows: (1) tumors confined to the same or different unilateral lung lobes and (2) tumors confined to different bilateral lung lobes. Moreover, the IPM group was classified into 3 subgroups based on the primary tumor size as follows: ≤3 cm subgroup; 3-5 cm subgroup; >5 cm subgroup.

The study was approved by the institutional review board of the Fifth Affiliated Hospital of Sun Yat-sen University (IRB protocol number ZDWY.FZYX.002). All the included patients provided signed informed consent. The clinical trial registration number is NCT03679936. Baseline clinical characteristics, including sex, age, height, weight, smoking history, and tumor characteristics, were obtained from electronic medical records with permission.

### 2.2. Dynamic PET Data Acquisition and Reconstruction

**Figure 2:**
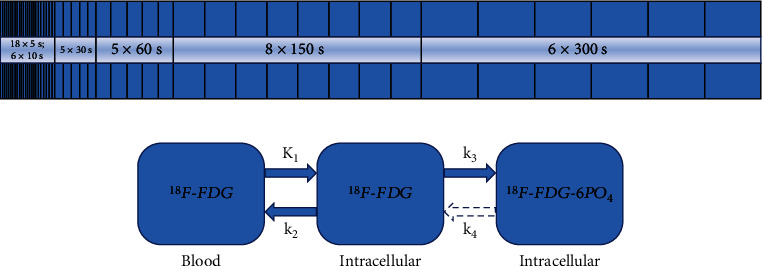
Dynamic acquisition mode and Patlak model. (a) Dynamic data were collected for 60 min comprising 48 frames: 18 × 5 s, 6 × 10 s, 5 × 30 s, 5 × 60 s, 8 × 150 s, and 6 × 300 s. (b) The Patlak plot has been developed for systems with irreversible trapping (*k*_4_ = 0). Most often it is applied for the analysis of FDG. *K*_1_ and *k*_2_ describe the ^18^F-FDG exchange between arterial plasma and tissue; *k*_3_ and *k*_4_ describe the exchange between ^18^F-FDG and ^18^F-FDG-6PO_4_ in tissue. As a result of unidirectional uptake of ^18^F-FDG, *k*_4_ = 0. *K*_*i*_ represents the ^18^F-FDG net uptake rate constant, i.e., *K*_*i*_ = *K*_1_*k*_3_/(*k*_2_ + *k*_3_).

Dynamic PET/CT scan was performed using 112-ring digital light guide PET/CT (uMI780, United Imaging, China). The patients were fasted for at least 6 h before scanning. The patient was restricted to movement in the scanner to avoid motion artifacts and conducive to subsequent accurate fusion of PET and CT. The scan covered the region between the thoracic inlet and the lower liver margin. Each PET/CT scan began with a transmission CT scan for 5 seconds that was used for attenuation correction. Next, an ^18^F-FDG bolus (range 143–327 MBq) was intravenously injected and a dynamic PET scan was acquired immediately as follows. Dynamic data were collected for 60 min comprising 48 frames with the following dimensions: 18 × 5 s, 6 × 10 s, 5 × 30 s, 5 × 60 s, 8 × 150 s, and 6 × 300 s (Figure 2(a)). The acquired data were corrected for decay, scatter, random, and attenuation; moreover, they were reconstructed using ordered subset expectation maximization.

### 2.3. PET Data Analysis

Two experienced nuclear medicine physicians analyzed the dynamic ^18^F-FDG PET/CT images using Carimas 2.10 software (Turku PET center, Finland). Reconstructed PET images were analyzed as follows: (1) definition of volumes of interest (VOIs); (2) obtaining the time-activity curve (TAC) of the left ventricle (i.e., plasma input function) and lung cancer nodules; (3) *K*_*i*_ calculation; (4) SUV_max_ calculation.

First, three-dimensional VOIs were manually drawn over the left ventricle (arterial blood pool) and tumors with a landmark using the Carimas 2.10 software. VOIs were visually localized using CT images. Moreover, the maximum diameters (*D*_max_) of the two tumors (the largest and second-largest lesion) were measured on multiplanar reconstructed CT images in the lung window via unidimensional measurements based on Response Evaluation Criteria in Solid Tumors 1.0 (RECIST criteria 1.0) [29].

Second, by projecting the VOIs onto the complete dynamic dataset, the Carimas analysis software automatically output relative TAC data of the VOIs. The left ventricle TAC was used for plasma input function, also known as image-derived input function (IDIF).

Third, the TAC of the left ventricle and tumors were used to fit standard Patlak modeling to assess FDG tissue kinetics via the least square regression method using Matlab 2018b (MathWorks Inc., Natick, MA, USA) [25, 26]. Patlak analysis was performed using data obtained between 20 and 60 postinjection minutes (i.e., between frames 38 and 48). The Patlak model for ^18^F-FDG metabolism in lung cancer (Figure 2(b)) has been previously described in detail [30].

Fourth, the last frame (at 55-60 min postinjection) of the dynamic scans was used for static analysis to obtain the SUV. The SUV_max_ of each pulmonary malignant lesion on PET/CT was extracted from the dynamic data.

### 2.4. Calculation of Indicators: ΔSUV_max_/*D*_max_ and Δ*K*_*i*_/*D*_max_

Based on SUV_max_ measured from ^18^F-FDG PET, the fitted *K*_*i*_ value obtained through Patlak graphic analysis, and the corresponding maximum diameter (*D*_max_) of two tumors, the absolute between-tumor difference of SUV_max_/*D*_max_ and *K*_*i*_/*D*_max_ (ΔSUV_max_/*D*_max_; Δ*K*_*i*_/*D*_max_) was calculated in both the sMPLC and IPM groups.

### 2.5. Histopathologic Analysis and Genetic Mutation Analysis

All the punctured tissue and resected specimens were fixed, dehydrated, embedded, sectioned, and stained for microscopic examination. Two experienced pathologists recorded the histopathologic diagnoses and features, including the histotype; lymphatic, nerve, and pleural invasion; extranodal extension; and regional lymph node metastasis.

Gene sequencing of some patients (18 out of 19 in sMPLC group; 20 out of 26 in IPM group) was carried out by the Beijing Genomics Institute. Gene sequencing projects include (1) Oseq TM-T tumor individualized diagnosis and treatment gene detection: 508 gene coding regions and partial intron regions closely related to solid tumors were detected; (2) Oseq TM-ctDNA noninvasive tumor individualized diagnosis and treatment gene detection: 508 gene coding regions and partial intron regions closely related to solid tumors were detected; (3) Oseq TM-T lung cancer individualized diagnosis and treatment gene detection: 20 specific gene mutations (i.e., ALK, EGFR, KRAS, BRAF, ERBB2, RET, MET, ROS1, NRAS, HRAS, DDR2, PIK3CA, AKT1, FBXW7, MAP2K1, FGFR3, NTRK1, KIT, PTEN, and TP53) associated with individualized drug use in lung cancer were detected.

### 2.6. Statistical Analysis

Between-group differences in baseline characteristics were assessed using an unpaired two-tailed Student's *t*-test or chi-square test. Quantitative data were analyzed through descriptive statistics (median (minimum–maximum)), scatter plots, Mann–Whitney test, and Pearson correlation (GraphPad Prism 8 software; two-tailed; 95% confidence intervals). Statistical significance was set at *p* < 0.05.

## 3. Results

### 3.1. Patient and Tumor Characteristics and Gene Detection Results

There were no differences in the baseline characteristics between the sMPLC and IPM groups.  Table 1 summarizes the gender distribution, age, height, body weight, FDG injection dose, and smoking history. IPM diagnoses were mainly based on radiologic and clinical patterns although the primary tumor was pathologically confirmed. sMPLC diagnoses were primarily based on histopathologic features. In both the sMPLC and IPM groups, the most commonly diagnosed tumor was adenocarcinoma with a majority being unilateral (68% and 71%, respectively).  Table 1 presents the tumor characteristics. Supplementary Tables S1 and S2, respectively, show the detailed gene mutation detection information of the sMPLC and IPM groups, mainly including sample type, tumor mutation burden, microsatellite instability, and mutations detected. EGFR mutations were the most common mutations whether in the sMPLC group or in the IPM group. Particularly, in the sMPLC group, almost two primary tumors were tested for mutation detection because of high resection rate and diagnosis requirement; however, only primary tumors were tested for mutation detection in the IPM group because almost only one lesion was resected or biopsied (Supplementary Tables S1 and S2).

### 3.2. sMPLC vs. IPM: Absolute Differences of SUV_max_/*D*_max_ or *K*_*i*_/*D*_max_

**Figure 3:**
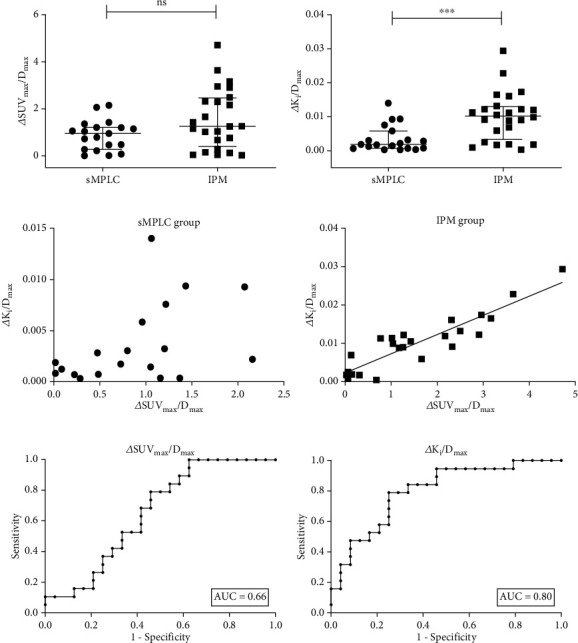
sMPLC vs. IPM: absolute differences of SUV_max_/*D*_max_ or *K*_*i*_/*D*_max_ and ROC curve analysis. (a, b) △*K*_*i*_/*D*_max_ was more sensitive than △SUV_max_/*D*_max_ in identifying sMPLC. Data are shown as median with interquartile range. Mann–Whitney test, ^∗∗∗^*p* < 0.001, ns = not significant. (c, d) Correlation between △SUV_max_/*D*_max_ and △*K*_*i*_/*D*_max_ in the sMPLC and IPM groups. (c) There was no correlation (Pearson *r* = 0.45, *p* > 0.05) between △SUV_max_/*D*_max_ and △*K*_*i*_/*D*_max_ in the sMPLC group. (d) There was a good correlation (Pearson *r* = 0.91, 95% CI: 0.79-0.96, *p* < 0.0001) between △SUV_max_/*D*_max_ and △*K*_*i*_/*D*_max_ in the IPM group. (e) ROC curve (AUC = 0.66, low diagnostic value) for △SUV_max_/*D*_max_. (f) ROC curve (AUC = 0.80, moderate diagnostic value) for △*K*_*i*_/*D*_max_. For the optimal cut-off value of △*K*_*i*_/*D*_max_ = 0.0059 with a sensitivity of 79% and specificity of 75%, the corresponding false-positive and false-negative rate was 25% and 21%.

**Figure 4:**
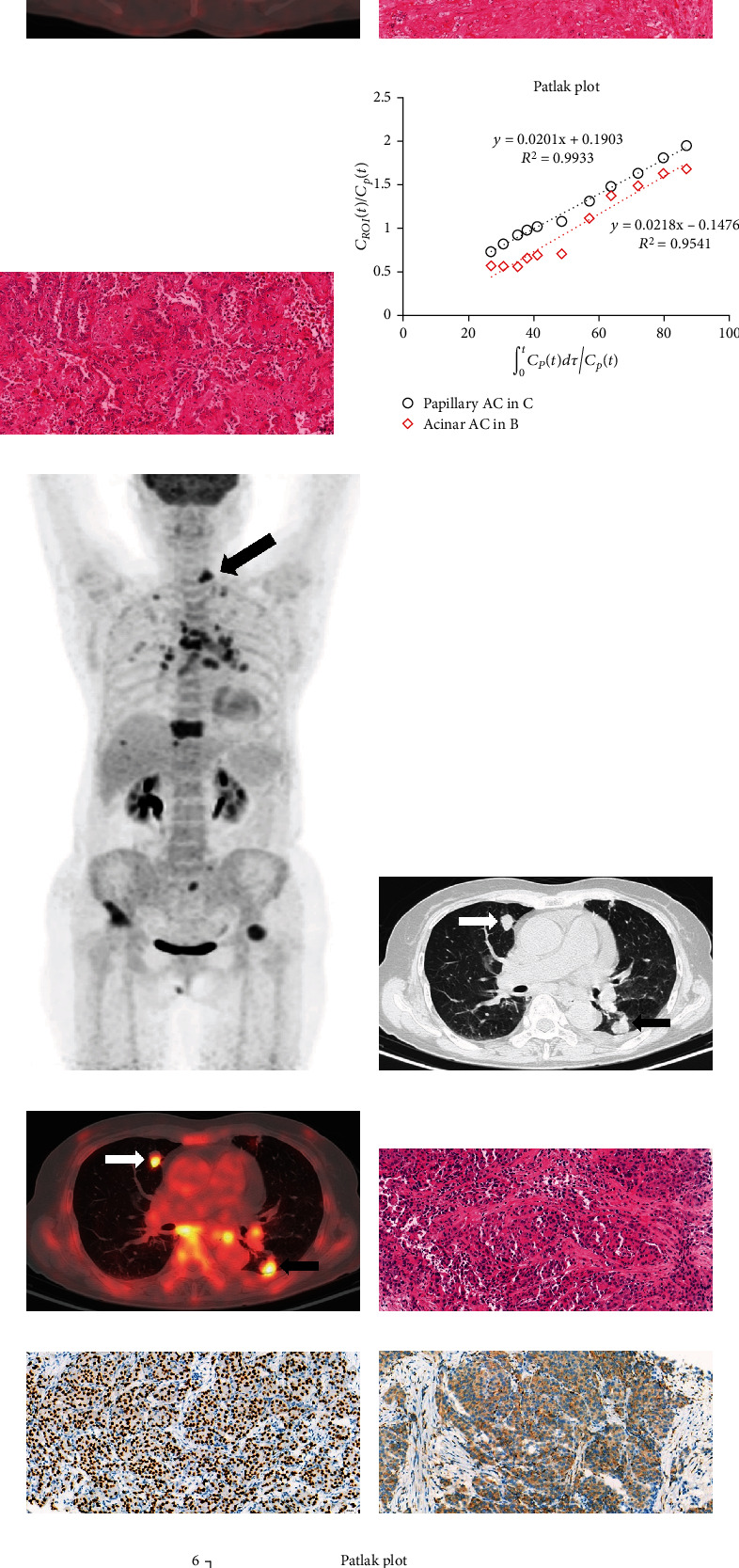
Representative sMPLC and IPM examples. (a–f) sMPLC examples. (a–c) FDG-PET of a patient (patient #1 in Table 3) with two synchronous primary lung tumors. Dynamic FDG PET/CT revealed an acinar adenocarcinoma in the right upper lobe (b) (white arrow, SUV_max_ = 2.72, *K*_*i*_ = 0.0218, *D*_max_ = 1.60 cm) and a synchronous papillary adenocarcinoma in the left upper lobe (c) (black arrow, SUV_max_ = 3.15, *K*_*i*_ = 0.0201, *D*_max_ = 1.80 cm). The values of △SUV_max_/*D*_max_ and △*K*_*i*_/*D*_max_ of both primary tumors were 0.05 and 0.0025, respectively. (d, e) HE staining. (f) The Patlak plot from lesions in (b) and (c), respectively. The slope of the Patlak plot represented the ^18^F-FDG net influx rate constant (*K*_*i*_, i.e. uptake rate constant). (g–m) IPM examples. (g–i) FDG-PET of a lung cancer patient (patient #24 in Table 4) with multiple intrapulmonary metastases. Dynamic FDG PET/CT demonstrated a lesion in the right upper lobe (h, i) (white arrow, SUV_max_ = 6.35, *K*_*i*_ = 0.0413, *D*_max_ = 1.60 cm) and another lesion in the left lower lobe (h, i) (black arrow, SUV_max_ = 6.88, *K*_*i*_ = 0.0456, *D*_max_ = 2.50 cm). The values of △SUV_max_/*D*_max_ and △*K*_*i*_/*D*_max_ of both tumors were 1.22 and 0.0076, respectively. Specifically, we only included and shown one lesion pair (two lesions: the largest and second-largest lesion) in this patient. (j–l) The HE and IHC (TTF-1 and Napsin A) staining of puncture biopsy tissue of left cervical lymph node ((g), black arrow), which indicated metastatic lung adenocarcinoma. (m) The Patlak plot from lesions in (h) and (i), respectively. According to the optimal cut-off value of △*K*_*i*_/*D*_max_ = 0.0059, the first patient was in agreement with pathological examination to be sMPLC, and the second patient was IPM. AC: adenocarcinoma; RUL: right upper lobe; LLL: left lower lobe.

There was no significant difference in ΔSUV_max_/*D*_max_ between the IPM and sMPLC groups (1.27 (0.03-4.72) vs. 0.96 (0.02-2.16), *p* > 0.05). Contrastingly, Δ*K*_*i*_/*D*_max_ was significantly higher in the IPM group than in the sMPLC group (0.0102 (0.0004-0.0294) vs. 0.0019 (0.0003-0.0140), *p* < 0.001) (Tables 2–4, Figures 3(a) and 3(b)). Supplementary Table S3 shows the comparison of ΔSUV_max_/*D*_max_ and Δ*K*_*i*_/*D*_max_ between different age groups and no significant difference was found within their respective groups. Considering a small clinical sample size, internal validation by Bootstrap method by SPSS 25.0 further validated the reproducibility. When performing bootstrapping based on 1000 bootstrap samples, the difference was still more significant in Δ*K*_*i*_/*D*_max_ (*p* = 0.003) than in ΔSUV_max_/*D*_max_ (*p* = 0.031) between two groups (Supplementary Table S4). However, we did not conduct external validation because of limited included cases at present. There was a good correlation (Pearson *r* = 0.91, 95% CI: 0.79-0.96, *p* < 0.0001, Figure 3(d)) between ΔSUV_max_/*D*_max_ and Δ*K*_*i*_/*D*_max_ in the IPM group but not the sMPLC group (Pearson *r* = 0.45, *p* > 0.05, Figure 3(c)). Figures 3(a)–3(d) present the data distribution and correlation in both groups while Figure 4 shows sMPLC and IPM examples. Tables 3 and 4 independently present individual results of each case in the sMPLC group and the IPM group including location of tumor, pathological subtype/type, 8th TNM stage, *D*_max_, SUV_max_, *K*_*i*_, ΔSUV_max_/*D*_max_, and Δ*K*_*i*_/*D*_max_. Specially, in the sMPLC group, each lesion was independent and staged separately.

### 3.3. Receiver Operating Characteristic (ROC) Curve Analysis

ROC curve analysis was used to determine the diagnostic accuracy of ΔSUV_max_/*D*_max_ and Δ*K*_*i*_/*D*_max_. For ΔSUV_max_/*D*_max_, ROC curve (AUC = 0.66, Figure 3(e)) indicated low diagnostic value. For Δ*K*_*i*_/*D*_max_, the area under the curve (AUC) was 0.80 (95% CI: 0.67–0.93, *p* < 0.001) for distinguishing sMPLC from IPM (Figure 3(f)), which suggested that Δ*K*_*i*_/*D*_max_ had a moderately high diagnostic value. The left upper corner (Δ*K*_*i*_/*D*_max_ = 0.0059) of the ROC curve with the maximal Youden index was chosen as the optimal cut-off point, which had a sensitivity and specificity of 79% and 75%, respectively. The corresponding false-positive and false-negative rate was 25% and 21%.

### 3.4. Unilateral sMPLC vs. Bilateral sMPLC

The lesion location has been reported to contribute to the over survival (OS) of MPLC [31, 32]; therefore, we compared the results between unilateral and bilateral sMPLC. The bilateral sMPLC group had a slightly higher Δ*K*_*i*_/*D*_max_. However, there was no significant difference between unilateral and bilateral sMPLC with respect to ΔSUV_max_/*D*_max_ and Δ*K*_*i*_/*D*_max_ (Supplementary Figures S1(a) and S1(b)).

### 3.5. Unilateral IPM vs. Bilateral IPM

According to the 8^th^ Edition Lung Cancer Stage Classification, unilateral and bilateral IPM could have different T and M categories, which indicates different stages and OS [33]. Therefore, we compared differences in the indicators between unilateral and bilateral IPM. The bilateral IPM group had a slightly higher ΔSUV_max_/*D*_max_. However, there was no significant difference between unilateral and bilateral sMPLC in terms of ΔSUV_max_/*D*_max_ and Δ*K*_*i*_/*D*_max_ (Supplementary Figures S1(c) and S1(d)).

### 3.6. IPM Subgroup Comparisons Based on Primary Tumor Size (≤3 cm vs. 3-5 cm vs. >5 cm)

Size plays a critical role in defining the T category as indicated by the proposed size cut-points of the 8^th^ edition Lung Cancer Stage Classification [34]. We compared differences in the indicators among three IPM subgroups (≤3 cm vs. 3-5 cm vs. >5 cm). Nevertheless, there was no significant among-group difference in ΔSUV_max_/*D*_max_ and Δ*K*_*i*_/*D*_max_ (Supplementary Figure S2).

### 3.7. Diagnostic Results Based on CT Characteristics

Table 5 summarizes the diagnostic results of at least two experienced radiologists based on CT characteristics in the sMPLC group and the IPM group. Based on CT characteristics, 26 out of 43 cases were diagnosed correctly. Based on the optimal cut-off value of △*K*_*i*_/*D*_max_ = 0.0059, 35 out of 43 cases were diagnosed correctly. Therefore, △*K*_*i*_/*D*_max_ performed better than experienced CT diagnosis in differentiating sMPLC from IPM.

## 4. Discussion

To our knowledge, this was the first study on the application of dynamic ^18^F-FDG PET to discriminate between tumors with common (i.e., IPM) and separate (i.e., sMPLC) lineages. Previous PET studies have predominantly focused on static imaging. Our findings indicated that dynamic ^18^F-FDG PET could be able to provide more detailed parameters for sMPLC identification.

The Martini and Melamed criteria are currently the most widely accepted; however, there remains no standard and uniform clinical guidelines for MPLC. There is no consensus among major lung cancer research institutes regarding MPLC classification. It is difficult to discriminate sMPLC from IPM without a lung, lymph node biopsy, or surgical procedure, which is important since the two conditions have significantly different therapeutic regimens and prognosis. Previous studies have reported that the OS of patients with MPLC was significantly better than those with metastatic tumors [31, 35]. Patients with MPLC are generally treated with curative surgical treatment while those in advanced stages receive chemotherapy or radiotherapy with palliative intent [2].

In routine clinical settings, preoperative differentiation of primary tumors from metastases is more critical than postoperative differentiation. Moreover, preoperative imaging examination plays a crucial role. It is recommended that patients with multiple pulmonary nodules undergo PET scans for careful systemic assessment based on the ACCP guidelines [2]. There is limited information regarding the imaging and metabolic characteristics of multiple lung cancer nodules [8]. Moreover, there have been few studies on the value of preoperative imaging for distinguishing MPLC from IPM in patients with multiple lung cancers [36].

Several case reports have reported incidental detection of sMPLC using ^18^F-FDG PET [37–40]. Contrastingly, there have been few studies indicating that ^18^F-FDG PET could be able to locate the clonal origin of synchronous multiple tumors [41–43]. Dijkman et al. reported that the relative between-tumor difference in SUV–ΔSUV%([bigger SUV − smaller SUV]/bigger SUV) could distinguish advanced disease from second primary tumors in patients with synchronous pulmonary lesions [41]. In this previous study, the second primary tumor group included initial primary cancers originating from the lung or other organs with second primary lung cancer. The metastatic disease group (control) included primary lung cancers with intrapulmonary metastases and extrapulmonary cancers with pulmonary metastases. Therefore, their findings reflect the differential diagnostic ability of *Δ*SUV% from a wider scope that includes cancer originating from the lungs or other organs. Furthermore, Pang et al. reported that ^18^F-FDG PET/CT could diagnose synchronous multiple primary cancers; further, *Δ*SUV% could identify the different pathological origins of synchronous cancers. Consistent with the findings by Dijkman et al., Pang et al. assessed synchronous multiple primary cancers, including primary lung cancers and extrapulmonary cancers [39]. Kosaka et al. assessed 75 cases of lung cancers with 296 metastases and reported that the SUV ratio (i.e., metastatic SUV to primary SUV) could differentiate primary lung cancer from metastasis [42]. However, most of the metastatic lesions were not pathologically confirmed and they only assessed lung cancers with metastases in the following sites: lymph nodes, bones, liver, pleura/lung, adrenal, kidney, and small intestine. Since this study did not involve a control group, its conclusions were empirical and probabilistic.

Similar to our study, Liu et al. only included patients with both cancers located in the lung and reported significant differences in the SUV_max_ ratio (bigger SUV_max_/smaller SUV_max_) between sMPLC and IPM. This indicated that the between-tumor SUV_max_ ratio could differentiate sMPLC from IPM [43]. In our study, we did not find a significant between-group difference in the SUV_max_ ratio and *K*_*i*_ ratio. These inconsistent findings could be attributed to differences in the sample size and study population, as well as inclusion and exclusion criteria.

Both Dijkman et al. and Liu et al. suggested that the SUV of tumors with a common lineage were more consistent than those with separate lineage [41, 43]. Moreover, they reported that related indicators (e.g., *Δ*SUV% and SUV_max_ ratio) were significantly higher in the sMPLC group than in the IPM group. Inconsistent with these previous findings, we found that ΔSUV_max_/*D*_max_ was numerically, but not significantly, larger in the IPM group than in the sMPLC group. Nevertheless, Δ*K*_*i*_/*D*_max_ was significantly larger in the IPM group than in the sMPLC group.

In our study, there was a good correlation between ΔSUV_max_/*D*_max_ and Δ*K*_*i*_/*D*_max_ in the IPM group, but not in the sMPLC group, which could be attributed to the identical and separate clone origin, respectively. The moderately high accuracy of Δ*K*_*i*_/*D*_max_ indicated by the AUC provided further evidence for the application of dynamic ^18^F-FDG PET as an auxiliary modality for discriminating sMPLC from IPM. We considered the size and SUV_max_ of the tumor as indexes since related studies have reported that these indexes can predict postoperative outcomes in patients with sMPLC [44]. Previous studies have reported adenocarcinoma as the major histopathological type in patients with sMPLC [45]. Moreover, the histologic type of initial and second primary lung cancers has been reported to be mostly similar [2, 35], which is consistent with our findings. Jiang et al. reported no significant OS differences between unilateral and bilateral MPLC [31]. Contrastingly, Trousse et al. reported that patients with bilateral MPLC had a better outcome than those with unilateral MPLC. Further, in patients with unilateral MPLC, lesions within the same lobe have been associated with better survival compared to those in different lobes [32]. Consequently, we assessed differences between unilateral and bilateral sMPLC. However, there was no significant between-group difference in ΔSUV_max_/*D*_max_ and Δ*K*_*i*_/*D*_max_. But the bilateral sMPLC group had a slightly higher Δ*K*_*i*_/*D*_max_ which may indicate a later disease stage.

>According to the 8^th^ Edition Lung Cancer Stage Classification [46], a separate T, N, M category should be designated to each tumor for second primary lung cancer. However, for patients with IPM, tumor nodules located in the same and different lobes of the unilateral lung and in the bilateral lung are staged as T3, T4, and M1a, respectively. As a result, unilateral IPM and bilateral IPM have different T and M categories, which is indicative of different stages. Regarding the site of the separate tumor nodule relative to the primary tumor in clinically staged patients, tumors in the same lobe are associated with a superior OS than those in different ipsilateral lobes. Moreover, the tumors with different contralateral lobes were associated with the worst OS [33]. Consequently, we determined differences in the indicators between unilateral IPM and bilateral IPM. Although there was no significant difference, the bilateral IPM group had a slightly higher ΔSUV_max_/*D*_max_ than the unilateral IPM group, which was indicative of a later disease stage. Size plays a significant role in defining the T category as shown by the proposed size cut-points of the 8^th^ Edition Lung Cancer Stage Classification. Survival analysis with 1 cm increments in tumor size showed that survival progressively decreased for each 1 cm cut-point [34]. The 3 cm, 5 cm, and 7 cm cut-points significantly separate T1, T2, T3, and T4, respectively, when only the primary tumor size is taken into consideration [46]. We did not observe a significant difference in ΔSUV_max_/*D*_max_ and Δ*K*_*i*_/*D*_max_ among the three IPM subgroups (≤3 cm vs. 3-5 cm vs. >5 cm) based on the primary tumor size.

We observed that Δ*K*_*i*_/*D*_max_ was a sensitive indicator, which could be explained by three main differences between SUV and Patlak *K*_*i*_. First, SUV measures phosphorylated and unphosphorylated FDG in the tumor while Patlak *K*_*i*_ only considers phosphorylated FDG [47]. Second, SUV is strongly dependent on uptake time and changes in plasma FDG clearance while Patlak *K*_*i*_ is independent of both [27, 47]. Third, Patlak *K*_*i*_ employs the integral under the plasma input function for normalization while SUV approximates this integral using the injected dose divided by the body weight [47]. Consequently, SUV and Patlak *K*_*i*_ measure different quantities with the later providing more robust dynamic information and more accurate ^18^F-FDG metabolism quantification. FDG metabolism quantification via Patlak analysis has several advantages over using SUV since it involves linear modeling without noise amplification and is independent of the uptake time and changes in plasma FDG clearance [27, 28]. However, for calculating fitted *K*_*i*_ by dynamic mathematical model, dynamic scan (tissue TAC) and arterial blood sampling (arterial blood TAC, but substitutable with IDIF) are required, which is unsuitable in clinical settings. The reason why it is difficult to perform dynamic PET/CT acquisition in the current clinical practice mainly include the following. (1) Long acquisition time and tedious acquisition process may not be practical for clinical centers with a large number of patients to be examined because of low working efficiency [13]. (2) Many patients, particularly those with advanced cancers, could not be able to tolerate too long acquisition time because the patient is restricted to movement in the scanner and will feel uncomfortable [13]. (3) The conventional and common PET scanner only has the limited axial field of view of about 15-25 cm, confining dynamic PET to a single bed position or one FOV [48]. On the other hand, static analysis only requires a 10-15 min static scan at a fixed time, which is usually 45-60 min after injection. In daily clinic, a single frame static image may provide enough desired information within acceptable error limits in most instances [13]. The selection of ideal analytical method should consider an optimal trade-off between quantitative accuracy and clinical convenience [13, 28]. Therefore, SUV is recommended when prioritizing clinical applicability and simplicity. Nevertheless, with the gradual development of the total-body PET scanner equipped with a long axial field of view of 194 cm, short-time dynamic total-body PET imaging with favorable spatial and temporal resolution and signal-to-noise ratio will make dynamic PET imaging more acceptable and popular in clinical application [48, 49].

This study has several limitations. Firstly, we employed a small sample size (43 cases); therefore, the results are preliminary and exploratory. Second, most of the cases with metastatic disease were diagnosed without histopathological confirmation based on typical clinical and radiological features, although the primary tumor was pathologically confirmed. Third, partial tumors were <1 cm with low SUV, which could have resulted in SUV biases due to the partial volume effect and statistical noise [50]. Fourth, for the optimal cut-off value of △*K*_*i*_/*D*_max_ = 0.0059 with a sensitivity of 79% and specificity of 75%, the corresponding false-positive and false-negative rate was 25% and 21% in this study. For this study, the diagnostic ability of a single indicator (△SUV_max_/*D*_max_ or △*K*_*i*_/*D*_max_) was not particularly high, but △*K*_*i*_/*D*_max_ performed better. Meanwhile, further external validation is needed.

## 5. Conclusions

Our findings indicated that dynamic ^18^F-FDG PET/CT could be a useful tool for distinguishing sMPLC from IPM complemented by histopathologic, clinical, and genetic evaluation, especially during preoperative assessment, which is mainly dependent on clinical and imaging characteristics. *K*_*i*_ calculation based on Patlak graphic analysis could be more sensitive for distinguishing metastatic disease from sMPLC in patients with multiple lung cancer nodules. There is a need for further studies to confirm the consistency of our findings.

## Figures and Tables

**Table 1:** Patient and tumor characteristics.

Category	sMPLC group (*n* = 19)		IPM group (*n* = 24)	*p* value
*Gender*				
Male	5		10	0.29
Female	14		14	
Age (mean ± SD) (years)	59 ± 9		56 ± 11	0.47
Height (mean ± SD) (cm)	160.50 ± 9.20		162.17 ± 10.79	0.60
Weight (mean ± SD) (kg)	58.16 ± 11.40		60 ± 12.63	0.62
FDG injection dose (mean ± SD) (MBq)	225.33 ± 41.07		224.22 ± 49.95	0.94
*Smoking history*				
Nonsmoker	15		16	0.62
Former smoker	1		3	
Current smoker	3		5	
*Conclusion mainly based on*				
Histopathologic subtype	12		0	
Gene mutation	1		0	
Clinical and radiologic features	6		24	
*Location*				
Unilateral	13		17	
Bilateral	6		7	
*Histopathology*	Primary lung tumor	Synchronous pulmonary tumor		
SCC	0	0	3	
AC	19	19	18	
Other	0	0	3	

sMPLC: synchronous multiple primary lung cancer; IPM: intrapulmonary metastases; SCC: squamous cell carcinoma; AC: adenocarcinoma; SD: standard deviation of the mean.

**Table 2:** Statistical results of the sMPLC and IPM groups.

		sMPLC group (*n* = 19)	IPM group (*n* = 24)	*p* value
Tumor 1	*D*_max−1_ (cm)	1.80 (0.50-5.50)	3.85 (1.60-8.80)	<0.0001
	SUV_max−1_	0.95 (0.32-8.76)	8.69 (1.33-16.06)	<0.0001
	*K*_*i*−1_	0.0028 (0.0004-0.0456)	0.0480 (0.0042-0.0756)	<0.0001
Tumor 2	*D*_max−2_ (cm)	0.90 (0.30-5.20)	0.85 (0.30-4.80)	0.71
	SUV_max−2_	0.80 (0.27-6.35)	1.03 (0.14-9.47)	0.23
	*K*_*i*−2_	0.0015 (0.0003-0.0413)	0.0028 (0.0002-0.0411)	0.37
Indicator	△SUV_max_/*D*_max_	0.96 (0.02-2.16)	1.27 (0.03-4.72)	0.08
	△*K*_*i*_/*D*_max_	0.0019 (0.0003-0.0140)	0.0102 (0.0004-0.0294)	<0.001

Data are shown as median (minimum–maximum). sMPLC: synchronous multiple primary lung cancer; IPM: intrapulmonary metastases; *D*_max−1_: largest diameter of tumor 1; *D*_max−2_: largest diameter of tumor 2; *K*_*i*_: influx rate constant; SUV_max_: maximum standardized uptake value; SUV_max−1_: SUV_max_ of tumor 1; SUV_max−2_: SUV_max_ of tumor 2; *K*_*i*−1_: *K*_*i*_ of tumor 1; *K*_*i*−2_: *K*_*i*_ of tumor 2; △SUV_max_/*D*_max_: absolute difference between SUV_max−1_/*D*_max−1_ and SUV_max−2_/*D*_max−2_; △*K*_*i*_/*D*_max_: absolute difference between *K*_*i*−1_/*D*_max−1_ and *K*_*i*−2_/*D*_max−2_.

**Table 3:** Individual results in the sMPLC group.

Patient no.	Location of tumor 1	Pathological subtype	8th TNM stage	*D*_max−1_ (cm)	SUV_max−1_	*K*_*i*−1_ (min^−1^)	Location of tumor 2	Pathological subtype	8th TNM stage	*D*_max−2_ (cm)	SUV_max−2_	*K*_*i*−2_ (min^−1^)	△SUV_max_/*D*_max_	△*K*_*i*_/*D*_max_
1	LUL	Papillary	pT1cN0M0, IA3	1.80	3.15	0.0201	RUL	Acinar	pT1bN2M0, IIIA	1.60	2.72	0.0218	0.05	0.0025
2	LUL	Invasive mucinous AC	pT1aN0M0, IA1	1.30	1.61	0.0192	LLL	Acinar	pT1aN0M0, IA1	1.50	0.27	0.0012	1.06	0.0140
3	LUL	Acinar	pT1bN0M0, IA2	1.20	0.32	0.0006	LLL	AC	pT1aN0M0, IA1	0.40	0.59	0.0015	1.20	0.0032
4	RUL	Acinar	pT1cN0M0, IA3	2.20	2.87	0.0089	RML	Lepidic	pT1aN0M0, IA1	0.80	0.41	0.0009	0.80	0.0030
5	RUL	Acinar	pT1cN0M0, IA3	1.80	1.67	0.0068	RLL	Lepidic	pT1bN0M0, IA2	1.00	0.91	0.0019	0.02	0.0019
6	RUL	Minimally invasive AC	pT1bN0M0, IA2	1.50	0.82	0.0006	LUL	Acinar	pT1cN0M0, IA3	2.80	1.59	0.0034	0.02	0.0008
7	LUL	Acinar	pT2bN0M0, IIA	5.50	1.47	0.0024	RUL	Papillary	pT2aN2M0, IIIA	5.20	3.85	0.0169	0.47	0.0028
8	RUL	Lepidic	pT1bN0M0, IA2	0.90	0.46	0.0007	RUL	Acinar	pT1aN0M0, IA1	0.90	0.89	0.0014	0.48	0.0007
9	RUL	Papillary	pT1bN0M0, IA2	1.20	0.62	0.0049	RLL	Acinar	pT1aN0M0, IA1	0.30	0.80	0.0006	2.16	0.0022
10	RLL	Acinar	pT1bN0M0, IA2	0.70	0.66	0.0011	RUL	Minimally invasive AC	pT1aN0M0, IA1	0.30	0.69	0.0004	1.37	0.0004
11	RUL	Acinar	pT2aN2M0, III A	3.00	8.76	0.0312	LLL	—	cT1aNxM0	1.00	0.84	0.0012	2.08	0.0093
12	RUL	Acinar	pT1bN0M0, IA2	1.20	0.62	0.0006	RLL	Papillary	pT1aN0M0, IA1	0.60	0.88	0.0038	0.96	0.0058
13	RUL	Minimally invasive AC	pT1aN0M0, IA1	0.60	0.95	0.0004	RLL	Acinar	pT1bN0M0, IA2	1.30	0.56	0.0004	1.16	0.0004
14	LLL	Acinar	pT1cN0M0, IA3	2.00	4.10	0.0206	RML	—	cT1aN0M0, IA1	0.60	0.37	0.0006	1.43	0.0094
15	RLL	Acinar	pT1cN0M0, IA3	3.10	1.65	0.0071	RUL	—	cT1aN0M0, IA1	0.50	0.79	0.0019	1.05	0.0014
16^a^	RML	Acinar	pT1aN0M0, IA1	2.00	0.48	0.0014	RLL	Acinar	pT2aN0M0, IB	2.90	0.95	0.0055	0.09	0.0012
17	RUL	—	cT1cN0M0, IA3	2.60	3.25	0.0120	RLL	AC	cT2bN0M0, IIA	4.60	4.45	0.0224	0.28	0.0003
18	RUL	Acinar	pT1bN0M0, IA2	2.00	0.93	0.0028	RLL	Minimally invasive AC	pT1aN0M0, IA1	0.50	0.60	0.0015	0.73	0.0017
19	LUL	—	cT1aN0M0, IA1	0.50	0.42	0.0005	RLL	Acinar	pT1aN0M0, IA1	0.90	0.55	0.0003	0.22	0.0007
Median (min–max)													0.96 (0.02-2.16)	0.0019 (0.0003-0.0140)

^a^Specifically, tumor 1 and tumor 2 in patient no. 16 have the same pathological subtype, but the different gene mutation. sMPLC: synchronous multiple primary lung cancer; *D*_max−1_: largest diameter of tumor 1; *D*_max−2_: largest diameter of tumor 2; *K*_*i*_: influx rate constant; SUV_max_: maximum standardized uptake value; SUV_max−1_: SUV_max_ of tumor 1; SUV_max−2_: SUV_max_ of tumor 2; *K*_*i*−1_: *K*_*i*_ of tumor 1; *K*_*i*−2_: *K*_*i*_ of tumor 2; △SUV_max_/*D*_max_: absolute difference between SUV_max−1_/*D*_max−1_ and SUV_max−2_/*D*_max−2_; △*K*_*i*_/*D*_max_: absolute difference between *K*_*i*−1_/*D*_max−1_ and *K*_*i*−2_/*D*_max−2_; RUL: right upper lobe; RML: right middle lobe; RLL: right lower lobe; LUL: left upper lobe; LLL: left lower lobe; AC: adenocarcinoma. Blank represents not available or there is no corresponding detection.

**Table 4:** Individual results in the IPM group.

Patient no.	Location of primary tumor	Pathological type	8th TNM stage	*D*_max−pt_ (cm)	SUV_max-pt_	*K*_*i*−pt_ (min^−1^)	Location of metastatic tumor	*D*_max−mt_ (cm)	SUV_max-mt_	*K*_*i*−mt_ (min^−1^)	△SUV_max_/*D*_max_	△*K*_*i*_/*D*_max_
1	RCLC	AC	cT2bN3M1c, IVB	4.80	7.70	0.0375	LUL	0.60	1.05	0.0036	0.15	0.0019
2	LUL	AC	cT4N2M1c, IVB	1.90	8.71	0.0687	LLL	0.70	5.24	0.0339	2.91	0.0122
3	RLL	AC	cT1cN3M1c, IVB	2.30	16.06	0.0620	LUL	0.30	1.00	0.0012	3.65	0.0228
4	LLL	AC	cT3N2M1b, IVA	6.20	14.04	0.0612	LLL	1.00	3.29	0.0212	1.02	0.0113
5	LUL	AC	cT1bN1M1c, IVB	1.60	8.84	0.0489	RML	0.60	0.48	0.0007	4.72	0.0294
6	LUL	AC	pT3N2M1b, IVA	2.90	6.46	0.0344	LUL	1.50	1.20	0.0020	1.43	0.0105
7	RCLC	AC	pT2aN3M1c, IVB	3.80	5.05	0.0263	RLL	4.00	5.52	0.0316	0.05	0.0010
8	LLL	NSCLC	cT4N3M1c, IVB	4.80	8.68	0.0458	LUL	1.30	4.51	0.0201	1.66	0.0060
9	LUL	Poorly differentiated carcinoma	cT4N3M1a, IVA	7.60	11.11	0.0556	RUL	2.50	9.47	0.0411	2.33	0.0091
10	LLL	AC	cT4N3M1c, IVB	5.80	8.06	0.0611	LUL	0.40	0.61	0.0014	0.13	0.0069
11	LCLC	AC	cT4N2M1a, IVA	8.80	7.89	0.0471	LLL	4.80	5.80	0.0341	0.31	0.0018
12	RCLC	SCC	cT2aN3M1b, IVA	3.90	7.26	0.0453	RUL	1.40	0.96	0.0039	1.17	0.0088
13	RLL	Invasive AC, acinar	pT1cN3M1c, IVB	3.00	10.22	0.0499	LUL	1.80	0.43	0.0002	3.17	0.0165
14	RUL	Lung cancer	cT3N3M1c, IVB	4.10	13.85	0.0551	RUL	0.50	0.60	0.0007	2.17	0.0120
15	RUL	Invasive AC, papillary	pT4N2M1a, IVA	2.20	4.98	0.0289	RLL	0.50	0.75	0.0009	0.77	0.0113
16	RML	AC	pT4N2M1b, IVA	3.30	9.49	0.0423	RLL	0.40	0.64	0.0003	1.27	0.0122
17	RCLC	AC	pT2bN1M1a, IVA	4.40	13.69	0.0756	LUL	1.10	2.28	0.0081	1.04	0.0099
18	RML	SCC	cT4N3M1a, IVA	4.90	9.79	0.0547	RLL	0.50	0.37	0.0011	1.26	0.0090
19	RML	AC	cT4N3M1c, IVB	6.00	6.97	0.0381	RUL	1.40	1.59	0.0065	0.03	0.0017
20	LLL	SCC	cT3N1M0, IIIA	5.00	9.85	0.0535	LLL	1.50	6.71	0.0359	2.50	0.0132
21	RLL	Invasive AC, acinar	pT4N0M0, IIIA	1.80	1.33	0.0042	RUL	0.30	0.43	0.0006	0.68	0.0004
22	LUL	Invasive AC, acinar	pT4N2M0, IIIB	2.80	9.26	0.0497	LLL	0.40	0.14	0.0002	2.96	0.0174
23	RLL	AC	cT4N2M0, IIIB	1.80	6.28	0.0327	RUL	0.50	0.59	0.0010	2.31	0.0161
24	RUL	AC	cT1cN3M1c, IVB	1.60	6.35	0.0413	LLL	2.50	6.88	0.0456	1.22	0.0076
Median (min–max)											1.27 (0.03-4.72)	0.0102 (0.0004-0.0294)

IPM: intrapulmonary metastases; *D*_max−pt_: largest diameter of primary tumor; *D*_max−mt_: largest diameter of metastatic tumor; *K*_*i*_: influx rate constant; SUV_max_: maximum standardized uptake value; SUV_max-pt_: SUV_max_ of primary tumor; SUV_max-mt_: SUV_max_ of metastatic tumor; *K*_*i*−pt_: *K_i_* of primary tumor; *K*_*i*−mt_: *K*_*i*_ of metastatic tumor; △SUV_max_/*D*_max_: absolute difference between SUV_max−pt_/*D*_max−pt_ and SUV_max−mt_/*D*_max−mt_; △*K*_*i*_/*D*_max_: absolute difference between *K*_*i*−pt_/*D*_max−pt_ and *K*_*i*−mt_/*D*_max−mt_; RUL: right upper lobe; RML: right middle lobe; RLL: right lower lobe; LUL: left upper lobe; LLL: left lower lobe; RCLC: right central lung cancer; LCLC: left central lung cancer; AC: adenocarcinoma; SCC: squamous cell carcinoma; NSCLC: non-small cell lung cancer.

**Table 5:** Diagnostic results based on CT characteristics.

	Patient no.	Tumor 1	Tumor 2	△SUV_max_/*D*_max_	△*K*_*i*_/*D*_max_
sMPLC group	1	Primary	Primary	0.05	0.0025
	2	Primary	Unsure	1.06	0.0140
	3	Unsure	Unsure	1.20	0.0032
	4	Primary	Unsure	0.80	0.0030
	5	Primary	Primary	0.02	0.0019
	6	Primary	Primary	0.02	0.0008
	7	Primary	Primary	0.47	0.0028
	8	Unsure	Unsure	0.48	0.0007
	9	Primary	Unsure	2.16	0.0022
	10	Primary	Unsure	1.37	0.0004
	11	Primary	Primary	2.08	0.0093
	12	Primary	Unsure	0.96	0.0058
	13	Unsure	Primary	1.16	0.0004
	14	Primary	Primary	1.43	0.0094
	15	Primary	Primary	1.05	0.0014
	16	Primary	Primary	0.09	0.0012
	17	Primary	Primary	0.28	0.0003
	18	Primary	Unsure	0.73	0.0017
	19	Primary	Primary	0.22	0.0007
IPM group	1	Primary	Metastasis	0.15	0.0019
	2	Primary	Metastasis	2.91	0.0122
	3	Primary	Metastasis	3.65	0.0228
	4	Primary	Metastasis	1.02	0.0113
	5	Primary	Metastasis	4.72	0.0294
	6	Primary	Primary	1.43	0.0105
	7	Primary	Primary	0.05	0.0010
	8	Primary	Metastasis	1.66	0.0060
	9	Primary	Metastasis	2.33	0.0091
	10	Primary	Metastasis	0.13	0.0069
	11	Primary	Metastasis	0.31	0.0018
	12	Primary	Metastasis	1.17	0.0088
	13	Primary	Primary	3.17	0.0165
	14	Primary	Metastasis	2.17	0.0120
	15	Primary	Unsure	0.77	0.0113
	16	Primary	Unsure	1.27	0.0122
	17	Primary	Primary	1.04	0.0099
	18	Primary	Metastasis	1.26	0.0090
	19	Primary	Metastasis	0.03	0.0017
	20	Primary	Metastasis	2.50	0.0132
	21	Primary	Unsure	0.68	0.0004
	22	Primary	Unsure	2.96	0.0174
	23	Primary	Metastasis	2.31	0.0161
	24	Primary	Metastasis	1.22	0.0076

“Primary” represents that tumors were considered as primary tumors. “Metastasis” represents that tumors were considered as metastatic tumors. “Unsure” represents that no definite diagnosis could be made based on CT characteristics.

## Data Availability

The data presented in this study are available on reasonable request from the corresponding author. The data are not publicly available due to privacy or ethical issues.

## Supplementary Materials

Supplementary Materials Figure S1: there was no significant difference in △SUV_max_/*D*_max_ and △*K*_*i*_/*D*_max_ between the unilateral and bilateral sMPLC group (a, b), as well as the IPM group (c, d). Figure S2: IPM subgroup comparisons based on primary tumor size (≤3 cm vs. 3-5 cm vs. >5 cm). Table S1: individualized diagnosis and treatment gene detection of the sMPLC group. Table S2: individualized diagnosis and treatment gene detection of the IPM group. Table S3: comparison of △SUV_max_/*D*_max_ and △*K*_*i*_/*D*_max_ between different age groups. Table S4: internal validation by Bootstrap.
