# Supplementary material for: Application of Dynamic 18F-FDG PET/CT for Distinguishing Intrapulmonary Metastases from Synchronous Multiple Primary Lung Cancer
Source: Mol Imaging. 2022 Jun 30;2022:8081299. doi: 10.1155/2022/8081299 (PMC9281433; doi:10.1155/2022/8081299)
Supplement: Supplementary Materials — Figure S1: there was no significant difference in △SUVmax/Dmax and △Ki/Dmax between the unilateral and bilateral sMPLC group (a, b), as well as the IPM group (c, d). Figure S2: IPM subgroup comparisons based on primary tumor size (≤3 cm vs. 3-5 cm vs. >5 cm). Table S1: individualized diagnosis and treatment gene detection of the sMPLC group. Table S2: individualized diagnosis and treatment gene detection of the IPM group. Table S3: comparison of △SUVmax/Dmax and △Ki/Dmax between different age groups. Table S4: internal validation by Bootstrap. [file 8081299.f1.docx]

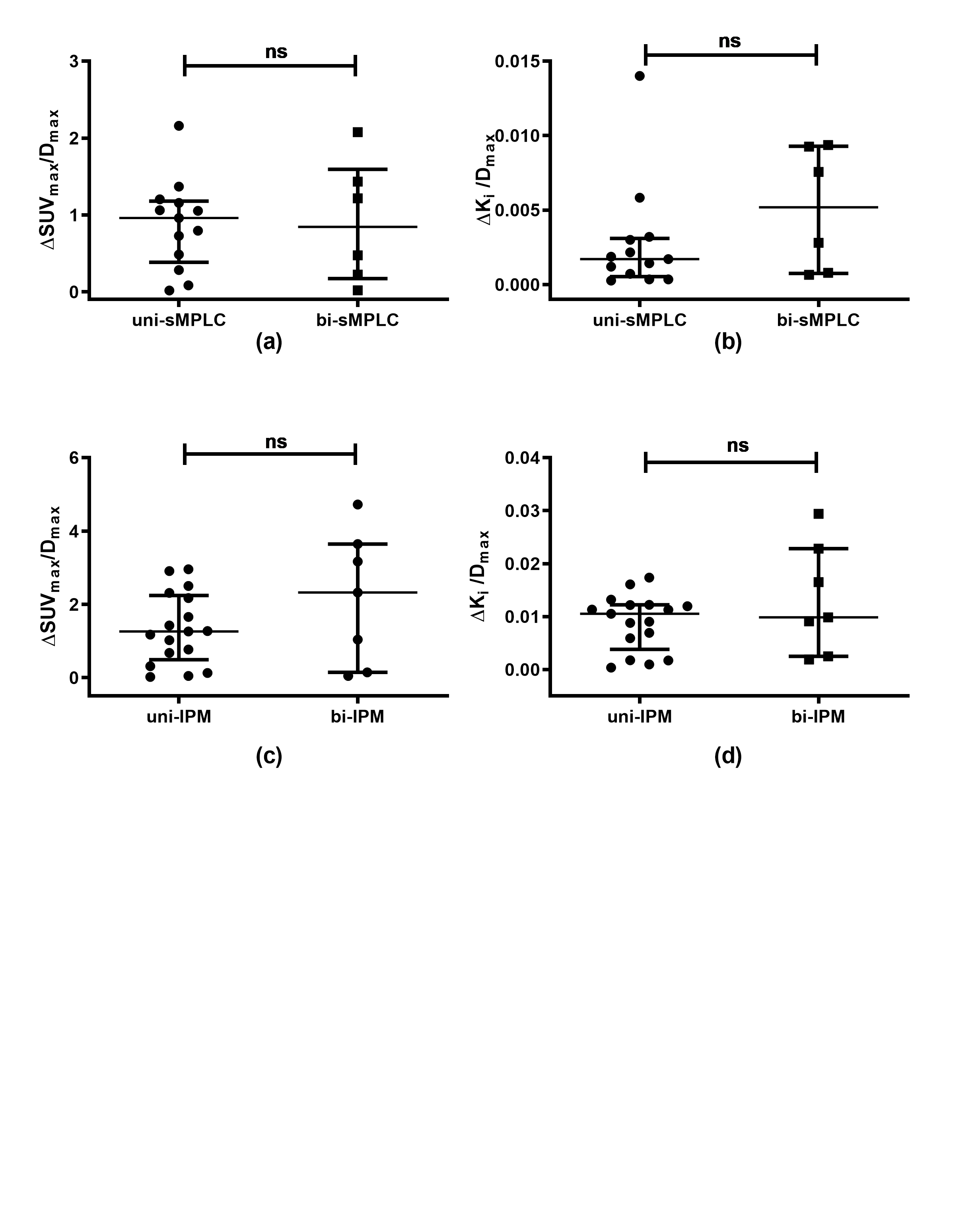


**Supplementary Figure S1. There was no significant difference in the △SUV_max_/D_max_ and △K_i_/D_max_ between the unilateral and bilateral sMPLC group (a-b), as well as the IPM group (c-d).** However, the ΔK_i_/D_max_ was slightly higher in the bilateral sMPLC group than in the unilateral sMPLC group. The ΔSUV_max_/D_max_ was slightly higher in the bilateral IPM group than in the unilateral IPM group. Data are shown as median with interquartile range. Mann-Whitney test, ns = not significant.


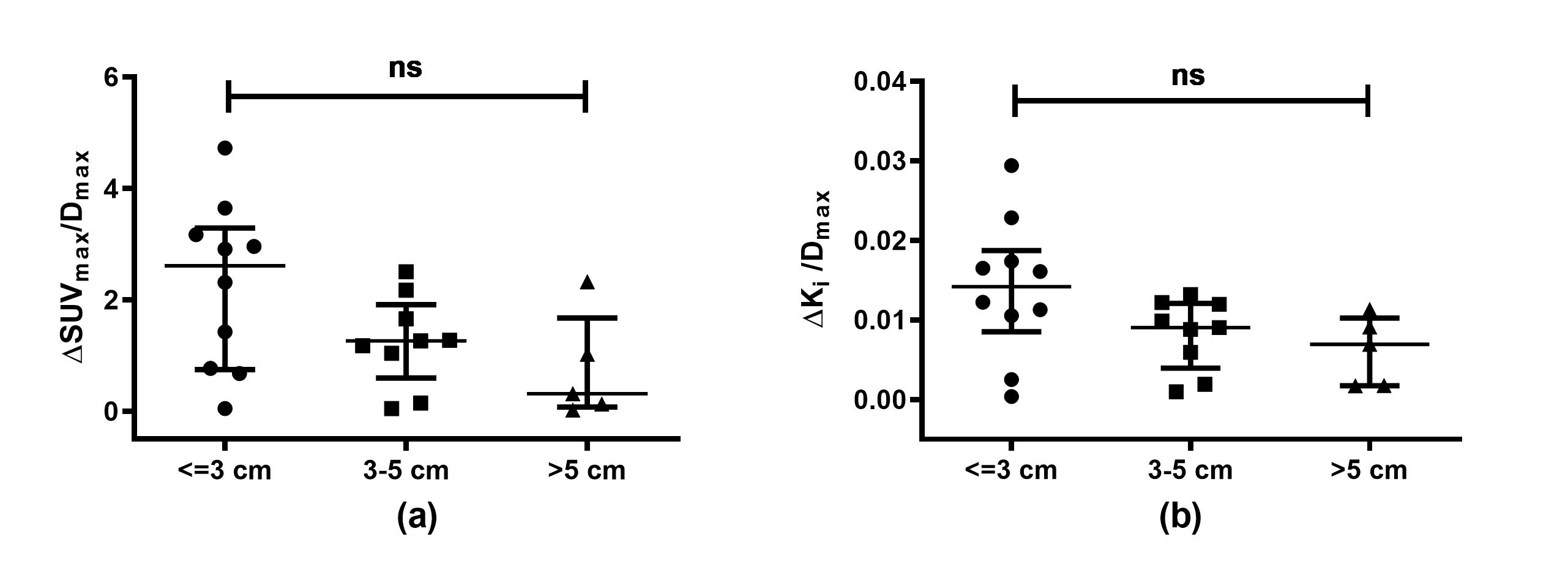


**Supplementary Figure S2. IPM subgroup comparisons based on primary tumor size (**≤ **3 cm vs. 3-5 cm vs. > 5 cm).**

There was no significant difference among three subgroups of IPM (primary tumor size ≤ 3 cm vs. 3-5 cm vs. >5 cm) in the ΔSUV_max_/D_max_ (a) and ΔK_i_/D_max_ (b). Data are shown as median with interquartile range. Kruskal-wallis test, ns = not significant.

**Supplementary** **Table S1. Individualized Diagnosis and Treatment Gene Detection of sMPLC Group**

|  | **Sample1** | | | | | **Sample2** | | |
| --- | --- | --- | --- | --- | --- | --- | --- | --- |
| **Patient No.** | **Sample type** | **Test** | **TMB (Muts / Mb)** | **MSI** | **Mutations detected** | **Sample type** | **Test** | **Mutations detected** |
| **1** | **Tissue** | **OseqTM-T+lung cancer** |  |  | **none** |  |  |  |
| **2** | **Tissue** | **OseqTM-T+lung cancer** |  |  | **CCDC6-RET** |  |  |  |
| **3** | **Tissue** | **OseqTM-T+lung cancer** |  |  | **EGFR exon21 L858R 5.7%** |  |  |  |
| **4** | **Tissue / hemocyte** | **OseqTM-Ttumor^a^** | **1.54** | **MSS** | **EGFR exon21 L858R 28.27%** |  |  |  |
|  |  |  |  |  | **TP53 exon5 P177L 28.46%** |  |  |  |
| **5** | **Tissue: RLL** | **OseqTM-T+lung cancer** |  |  | **none** | **Tissue: RUL** | **OseqTM-T+lung cancer** | **EGFR exon19 E746_A750del 11.18%** |
| **6** | **Tissue: RUL** | **OseqTM-T+lung cancer** |  |  | **EGFR exon21 L858R 7.39%** |  |  |  |
| **7** | **Tissue: RUL** | **OseqTM-T+lung cancer** |  |  | **EGFR exon21 L858R 29.63%** | **Tissue: LUL** | **OseqTM-T+lung cancer** | **none** |
| **8** | **Tissue: RUL** | **OseqTM-T+lung cancer** |  |  | **EGFR exon19 E746_T751delinsV 10.67%** | **Tissue: RUL** | **OseqTM-T+lung cancer** | **none** |
| **9** | **Tissue: RLL** | **OseqTM-T+lung cancer** |  |  | **EGFR exon21 L858R 4.91%** | **Tissue: RUL** | **OseqTM-T+lung cancer** | **none** |
| **10** | **Tissue** | **OseqTM-T+lung cancer** |  |  | **HRAS G13S 1.54%** |  |  |  |
|  |  |  |  |  | **EGFR exon19 L747_S752delinsQ H 1.15%** |  |  |  |
| **11** |  |  |  |  | **no tests** |  |  |  |
| **12** | **Tissue** | **OseqTM-T+lung cancer** |  |  | **EGFR exon21 L858R 13.29%** | **Tissue** | **OseqTM-T+lung cancer** | **none** |
| **13** | **Tissue** | **OseqTM-T+lung cancer** |  |  | **none** |  |  |  |
| **14** | **Tissue** | **OseqTM-T+lung cancer** |  |  | **KRAS G12C 20.34%** |  |  |  |
| **15** | **Tissue** | **OseqTM-T+lung cancer** |  |  | **EGFR exon21 L858R 20.93%** |  |  |  |
| **16** | **Tissue** | **OseqTM-T+lung cancer** |  |  | **EGFR exon19 L747_P753delinsS 25.02%** | **Tissue** | **OseqTM-T+lung cancer** | **EGFR exon19 L747_S752del 16.99%** |
| **17** | **Tissue** | **OseqTM-Ttumor^a^** | **6.45** | **MSS** | **KRAS exon2 G12A 43.26%** |  |  |  |
|  |  |  |  |  | **TP53 exon5 Y163C 26.49%** |  |  |  |
|  |  |  |  |  | **KRAS Copy number amplification 3.54%** |  |  |  |
| **18** | **Tissue** | **OseqTM-T+lung cancer** |  |  | **EGFR exon21 L858R 18.59%** |  |  |  |
| **19** | **Tissue** | **OseqTM-T+lung cancer** |  |  | **EGFR exon19 E746_S752delinsV 4.19%** |  |  |  |

^a^: Multiple somatic related gene mutations were detected, and only the most clinically significant mutations were shown.

TMB: Tumor mutation burden

MSI: Microsatellite instability

MSS: Microsatellite stable

RUL: right upper lobe; RML: right middle lobe; RLL: right lower lobe

LUL: left upper lobe; LLL: left lower lobe

Tissue: Formalin-fixed paraffin-embedded tissues

Blank represents not available or there is no corresponding detection

**Supplementary Table S2.** **Individualized Diagnosis and Treatment Gene Detection of IPM Group**

|  | **Sample** | | | | | | | | | |
| --- | --- | --- | --- | --- | --- | --- | --- | --- | --- | --- |
| **Patient No.** | **Sample type** | **Test** | **TMB (Muts / Mb)** | **MSI** | **Mutations detected** | | | | | |
|  |  |  |  |  | **Gene** | **Nucleotide variation** | **Amino acid variation** | **Gene subregion** | **Mutation frequency** | **Transcript** |
| **1** |  |  |  |  | **EGFR** |  |  | **EX19** |  |  |
| **2** | **Tissue / hemocyte** | **OseqTM-Ttumor^a^** | **7.69** | **MSS** | **KRAS** | **c.35G>T** | **p.G12V** | **EX2** | **32.95%** |  |
| **3** |  |  |  |  | **no tests** |  |  |  |  |  |
| **4** | **Tissue** | **OseqTM-T+lung cancer** |  |  | **EGFR** |  | **p.E746_A750del** | **EX19** | **67.05%** |  |
| **5** | **Tissue** | **OseqTM-T+lung cancer** |  |  | **EGFR** |  | **p.E746_A750del** | **EX19** | **1.23%** |  |
| **6** | **Plasma** | **OseqTM-ctDNA^a^** | **1.43** | **MSS** | **EGFR** | **c.2239_2240delTTinsCC** | **p.L747P** | **EX19** | **4.14%** | **NM_005228.3** |
|  |  |  |  |  | **BRCA2** | **c.6573_6589delAATGGAAATTGGTAAAA** | **p.K2191Nfs*2** | **EX11** | **2.27%** | **NM_000059.3** |
| **7** |  |  |  |  | **EGFR** | **c.2155G>A** | **p.G719S** |  |  |  |
|  |  |  |  |  | **EGFR** | **c.185T>G** | **p.L62R** |  |  |  |
|  |  |  |  |  | **TP53** | **c.814G>A** | **p.V272M** |  |  |  |
| **8** |  |  |  |  | **no tests** |  |  |  |  |  |
| **9** | **Tissue / hemocyte** | **OseqTM-Ttumor** | **1.03** | **MSS** | **PTEN** | **c.402_404del** | **p.M134del** | **EX5** | **7.16%** | **NM_000314.4** |
| **10** |  |  |  |  | **no tests** |  |  |  |  |  |
| **11** | **Tissue / hemocyte** | **OseqTM-Ttumor^a^** | **9.74** | **MSS** | **KRAS** | **c.34G>T** | **p.G12C** | **EX2** | **58.06%** | **NM_033360.2** |
|  |  |  |  |  | **PIK3CA** | **c.1633G>A** | **p.E545K** | **EX10** | **32.74%** | **NM_006218.2** |
|  |  |  |  |  | **NTRK3** | **c.1822G>T** | **p.G608C** | **EX16** | **21.84%** | **NM_002530.3** |
| **12** |  |  |  |  | **no tests** |  |  |  |  |  |
| **13** |  |  |  |  | **no tests** |  |  |  |  |  |
| **14** | **Plasma / hemocyte** | **OseqTM-ctDNA^a^** | **2.05** |  | **EGFR** | **c.2573T>G** | **p.L858R** | **EX21** | **24.60%** | **NM_005228.3** |
|  |  |  |  |  | **EGFR** | **c.2126A>G** | **p.E709G** | **EX18** | **0.46%** | **NM_005228.3** |
| **15** | **Tissue** | **OseqTM-T+lung cancer** |  |  | **EML4-ALK** |  |  |  |  |  |
| **16** | **Tissue / hemocyte** | **OseqTM-Ttumor^a^** | **12.31** | **MSS** | **TP53** | **c.919+1G>A** | **-** | **IVS8** | **18.94%** | **NM_000546.5** |
|  |  |  |  |  | **RET** | **c.1513G>C** | **p.E505Q** | **EX7** | **7.03%** | **NM_020975.4** |
| **17** | **Tissue** | **OseqTM-T+lung cancer** |  |  | **none** |  |  |  |  |  |
| **18** | **Tissue** | **OseqTM-T+lung cancer** |  |  | **none** |  |  |  |  |  |
| **19** |  |  |  |  | **no tests** |  |  |  |  |  |
| **20** | **Tissue** | **OseqTM-Ttumor^a^** | **6.67** | **MSS** | **EGFR** | **c.2126A>C** | **p.E709A** | **EX18** | **34.08%** | **NM_005228.3** |
|  |  |  |  |  | **EGFR** | **c.2573T>G** | **p.L858R** | **EX21** | **33.37%** | **NM_005228.3** |
| **21** | **Tissue** | **OseqTM-T+lung cancer** |  |  | **none** |  |  |  |  |  |
| **22** | **Tissue** | **OseqTM-T+lung cancer** |  |  | **none** |  |  |  |  |  |
| **23** | **Tissue / hemocyte** | **OseqTM-Ttumor^a^** | **8.72** | **MSI-L** | **TP53** | **c.838A>G** | **p.R280G** | **EX8** | **26.76%** | **NM_000546.5** |
|  |  |  |  |  | **MAP2K1** | **c.307_312del** | **p.I103_K104del** | **EX3** | **18.14%** | **NM_002755.3** |
| **24** | **Tissue** | **OseqTM-Ttumor^a^** | **0.36** | **MSS** | **EGFR** | **c.2237_2256delAATTAAGA GAAGCAACATCTinsTC** | **p.E746_S752delinsV** | **EX19** | **20.62%** | **NM_005228.3** |

^a^: Multiple somatic related gene mutations were detected, and only the most clinically significant mutations were shown

TMB: Tumor mutation burden

MSI: Microsatellite instability

MSS: Microsatellite stable

MSI-L: Low frequency MSI

Tissue: Formalin-fixed paraffin-embedded tissues

Blank represents not available or there is no corresponding detection

**Supplementary Table S3. Comparison of △SUVmax/Dmax and △Ki/Dmax between different age groups**

|  | sMPLC group | | IPM group | |
| --- | --- | --- | --- | --- |
|  | △SUV_max_/D_max_ | △K_i_/D_max_ | △SUV_max_/D_max_ | △K_i_/D_max_ |
| Age (﹤ 60) | 1.16 (0.22 - 2.16) | 0.0017 (0.0003 - 0.0094) | 0.91 (0.03 - 4.72) | 0.0090 (0.0004 - 0.0294) |
| Age ( ≥ 60) | 0.48 (0.02 - 2.08) | 0.0023 (0.0007 - 0.0140) | 1.54 (0.05 - 3.65) | 0.0116 (0.0018 - 0.0228) |
| *p* value | 0.07 | 0.48 | 0.27 | 0.27 |

**Supplementary Table S4. Internal validation by Bootstrap**

| Bootstrap for Independent Samples Test | | | | | | | |
| --- | --- | --- | --- | --- | --- | --- | --- |
|  |  |  | Bootstrap^a^ | | | | |
|  |  | Mean Difference | Bias | Std. Error | Sig. (2-tailed) | 95% Confidence Interval | |
|  |  |  |  |  |  | Lower | Upper |
| △SUV_max_/D_max_ | Equal variances assumed | -0.6881 | 0.0116 | 0.2850 | 0.0310 | -1.2781 | -0.1100 |
| △K_i_/D_max_ | Equal variances assumed | -0.0067 | < 0.0001 | 0.0017 | 0.0030 | -0.0100 | -0.0032 |
| ^a^ Unless otherwise noted, bootstrap results are based on 1000 bootstrap samples | | | | | | | |
